# Human epidermal growth factor receptor 3-targeted near-infrared photoimmunotherapy in a xenograft mouse model of breast cancer

**DOI:** 10.1186/s12885-026-16170-4

**Published:** 2026-05-18

**Authors:** Makoto Kano, Aki Furusawa, Hiroshi Fukushima, Seiichiro Takao, Shuhei Okuyama, Hiroshi Yamamoto, Motofumi Suzuki, Ko Kitamura, Miyu Kano, Peter L Choyke, Hisataka Kobayashi

**Affiliations:** Molecular Imaging Branch, Center for Cancer Research, National Cancer Institute, NIH, 10 Center Drive, Building 10, Room B3B47, Bethesda, MD 20892 USA

**Keywords:** Breast cancer, Human epidermal growth factor receptor 3 (HER3), Luminal breast cancer, Near-infrared photoimmunotherapy, Triple-negative breast cancer

## Abstract

**Background:**

Human epidermal growth factor receptor 3 (HER3), a member of the HER/ErbB family, is broadly expressed across major breast cancer subtypes and contributes to tumor initiation, progression, and therapy resistance. Although numerous HER3-directed therapies have been evaluated in preclinical and clinical settings, limited efficacy and safety concerns have prevented any from achieving clinical approval. Near-infrared photoimmunotherapy (NIR-PIT) is a modality that selectively kills antibody-bound target cells upon NIR irradiation. In this study, we evaluated HER3-targeted NIR-PIT in breast cancer cell lines in vitro and in xenograft mouse models in vivo.

**Methods:**

Triple-negative (MDA-MB-468) and Luminal (MCF-7) breast cancer cells were utilized. In vitro, the therapeutic efficacy of HER3-targeted NIR-PIT was assessed by monitoring morphological changes via differential interference contrast microscopy and evaluating cell cytotoxicity using propidium iodide staining. In vivo, MDA-MB-468 and MCF-7 were subcutaneously xenografted into nude mice. Mice bearing MDA-MB-468 tumors were assigned to Control, HER3 antibody-photoabsorber conjugate i.v., and HER3-targeted NIR-PIT groups, while MCF-7 tumor-bearing mice were divided into Control and HER3-targeted NIR-PIT groups. Tumor growth and survival rates were monitored, and histological alterations post-NIR-PIT were analyzed using Hematoxylin and eosin (H&E) and pan-cytokeratin immunohistochemical staining.

**Results:**

HER3-targeted NIR-PIT induced robust cancer cell death in vitro, significantly suppressed tumor growth and prolonged survival in vivo, and achieved complete responses in 30% of treated mice bearing triple-negative breast cancer xenografts.

**Conclusions:**

These findings support HER3-targeted NIR-PIT as a promising therapeutic strategy for HER3-positive breast cancer.

**Supplementary Information:**

The online version contains supplementary material available at 10.1186/s12885-026-16170-4.

## Background

Human epidermal growth factor receptor 3 (HER3/ErbB3) is a member of the HER/ErbB family, which also includes epidermal growth factor receptor (EGFR; HER1/ErbB1), HER2/ErbB2, and HER4/ErbB4 [1, 2]. Overexpression of EGFR, HER2, and HER3 has been associated with poor prognosis [3], and these proteins have been widely targeted in cancer therapy. Although HER3 has limited intrinsic kinase activity [4], it plays a critical role in cancer progression, metastasis, and drug resistance, primarily through heterodimerization with EGFR or HER2, which potently activates the PI3K/Akt signaling pathway [3, 5].

In breast cancer, reported HER3 expression rates vary widely (20%–70%) but are generally higher than those of other HER family members [6–12]. Notably, HER3 is broadly expressed across all major breast cancer subtypes—including luminal, HER2-positive, and triple-negative breast cancer (TNBC)—supporting its potential as a universal therapeutic target (Table 1) [8, 13–17].

**Table 1:** Summary of published data on ubiquitous expression of HER in breast cancer

Ref.	Expression Level	Detection Method	Cohort (*n*)	Subtype	EGFR expression	HER2 expression	HER3 expression	Notes	Ref.
Witton et al. 2003	Protein	IHC	220	All	16.40%	22.80%	17.50%	HER3 positive IHC score 3+	[6]
El-Rehim et al. 2004	Protein	IHC	1500	All	20.10%	31.80%	45.00%	tumours with 5% of the neoplastic cells	[7]
Youn Bae et al. 2013	Protein	IHC	950	All	13.68%	12.00%	52.11%	HER3 positive IHC score 1+, 2+, 3+	[8]
Berfhoff et al. 2014	Protein	IHC	110	All	N/A	15.45%	20.90%	HER3 positive IHC score 3+	[9]
Luhtala et al. 2018	Protein	IHC	308	All	N/A	57.45%	75.30%	HER3 positive IHC score 2+	[10]
Makoui et al. 2024	Protein	IHC	444	All	N/A	21.30%	30.10%	HER3 positive IHC score 2+, 3+	[11]
Lee et al. 2024	Protein	IHC	320	All	N/A	8.80%	47.81%	HER3 positive IHC score 3+	[12]
Youn Bae et al. 2013	Protein	IHC	607	Luminal			55.02%	HER3 positive IHC score 1+, 2+, 3+	[8]
Sinevici et al. 2024	Protein	WB	20	Luminal			40.00%		[13]
Youn Bae et al. 2013	Protein	IHC	114	HER2			63.15%	HER3 positive IHC score 1+, 2+, 3+	[8]
Czopek et al. 2013	Protein	IHC	35	HER2			45.71%	HER-3 positive cells > 50% of tumor cells	[14]
Dębska-Szmich et al. 2015	Protein	IHC	81	HER2			55.60%		[15]
Sinevici et al. 2024	Protein	WB	11	HER2			55.00%		[13]
Youn Bae et al. 2013	Protein	IHC	229	TNBC			38.86%	HER3 positive IHC score 1+, 2+, 3+	[8]
Ho-Pun-Cheung et al. 2020	Protein	IHC	51	TNBC			17.60%	H-Score > 10, EGFR 13.8%	[16]
Lyu et al. 2023	Protein	IHC	125	TNBC			49.60%	HER3 positive IHC score 1+, 2+, 3+	[17]

Summary of previously published studies evaluating EGFR, HER2, and HER3 expression in breast cancer, and HER3 expression across breast cancer subtypes. The table includes the detection method, breast cancer subtype, sample size, and reported frequency or level of HER expression

*IHC* immunohistochemistry, *EGFR* epidermal growth factor receptor, *HER* human epidermal growth factor receptor, *Ref.* Reference, *TNBC* triple-negative breast cancers, *WB* Western Blot

Therapeutic strategies targeting the extracellular domain of HER3 have been extensively developed, with a particular focus on engineering monoclonal antibodies (mAbs) to enhance therapeutic efficacy [18, 19]. For example, by targeting distinct epitopes, some mAbs are designed not only to block ligand binding directly but also to prevent the structural changes required for heterodimerization with EGFR or HER2 [20, 21]. Another key approach focuses on downregulation—inducing the cell to internalize and degrade the receptor, thereby reducing its presence on the cell surface [21, 22]. Researchers are also exploring antibody mixtures that target multiple sites to enhance inhibitory effects [23–26]. Despite these sophisticated designs, clinical efficacy has been limited, and no HER3-targeted therapy has yet received regulatory approval [3, 5]. This limited efficacy occurs because compensatory signaling through partner receptors allows tumor cells to maintain downstream activation even when specific pathways are blocked [27]. In this context, antibody–drug conjugates (ADCs) have emerged as a promising platform: by coupling a HER3-targeted antibody with a cytotoxic payload, ADCs can deliver potent chemotherapy directly to HER3-expressing tumor cells [28]. Although HER3-directed ADCs have shown encouraging antitumor activity in clinical trials, challenges remain, including enhancing ADC internalization and payload delivery in tumor cells while minimizing off-target toxicity in normal tissues [29, 30]. Consequently, there is a need for novel HER3-targeted approaches that operate through mechanisms distinct from conventional signaling inhibition.

Near-infrared photoimmunotherapy (NIR-PIT) is an innovative treatment that employs a monoclonal antibody (mAb) conjugated to a photoactivatable dye, IRDye700DX, to form an antibody–photoabsorber conjugate (APC). Upon exposure to near-infrared (NIR) light, NIR-PIT selectively destroys cells expressing the antigen targeted by the mAb [31, 32]. APCs can be directed against a variety of tumor-associated membrane antigens. For example, EGFR-targeted NIR-PIT has been approved in Japan for advanced head and neck cancers, which commonly overexpress EGFR [33]. In breast cancer, we previously reported that EGFR-targeted NIR-PIT is effective in EGFR-positive TNBC [34], and Yamashita et al. demonstrated the efficacy of HER2-targeted NIR-PIT in HER2-positive disease [35]. However, the efficacy of HER3-targeted NIR-PIT remains unknown.

In this study, we aimed to provide an initial proof-of-concept for the therapeutic efficacy of NIR-PIT targeting HER3-expressing tumor cells. Using xenograft models derived from luminal and TNBC cell lines, we evaluated the potential of this approach to achieve localized tumor control across different breast cancer molecular profiles.

## Methods

### Synthesis of IR700-conjugated anti-HER3 mAb

APC was synthesized as described previously [36]. In brief, 1 mg of anti-HER3 mAb (6.7 nmol, clone Patritumab, Abinvivo, Metuchen, NJ, USA) was incubated with a five-fold molar excess of IR700 NHS ester (10 mmol/L in DMSO, LI-COR Biosciences, Lincoln, NE, USA). The resulting anti-HER3 mAb APC was designated as HER3-IR700. To confirm successful conjugation of HER3-IR700, both the HER3-IR700 and the unconjugated control antibody were analyzed by sodium dodecyl sulfate-polyacrylamide gel electrophoresis (SDS-PAGE). Samples were loaded onto 4–20% Tris-Glycine gradient polyacrylamide gels (Cat# XP04200BOX, Thermo Fisher Scientific, Rockford, IL, USA) and electrophoresed at 80 V for 2.5 h. After electrophoresis, the gel was stained with Colloidal Blue and imaged under white light to visualize protein bands. The same gel was then examined using a Pearl Imager (LI-COR Biosciences).

### Tumor cell lines

A luciferase-expressing MDA-MB-468 (human TNBC, RRID: CVCL_0419, MDA-MB-468 Luc) cell line was obtained from the Division of Cancer Treatment and Diagnosis Tumor Repository, NCI Frederick (Frederick, MD, USA). MCF-7 (human luminal breast cancer, RRID: CVCL_0031) cell line was kindly provided by Dr. Raya Mandler (NCI/NIH, Rockville, MD, USA). MDA-MB-468 Luc cells were cultured in RPMI 1640 medium (Cat# 11875119, Thermo Fisher Scientific), and MCF-7 cells were cultured in EMEM medium (Cat# 30-2003, ATCC, Manassas, VA, USA), each supplemented with 10% fetal bovine serum (Cat# A5670801, Thermo Fisher Scientific), 100 IU/mL penicillin, and 100 µg/mL streptomycin (Cat# 15140122, Thermo Fisher Scientific), in a humidified incubator in an atmosphere of 95% air and 5% carbon dioxide at 37 °C. Both MDA-MB-468 Luc and MCF-7 cell lines were authenticated by IDEXX BioAnalytics (Westbrook, ME, USA) via STR profiling in August 2023.

### In vitro flow cytometric analysis of HER3 expression

A total of 2 × 10^5^ MDA-MB-468 Luc or MCF-7 cells were harvested and resuspended in 50 µL phosphate-buffered saline (PBS), and stained with 0.5 µL PE-conjugated anti-human HER3 Ab (clone 1B4C3, RRID: AB_2099569, BioLegend, San Diego, CA, USA) or the corresponding PE-conjugated isotype control (Mouse IgG2a, κ, RRID: AB_2800438, BioLegend), with a Fixable Viability Dye (Cat# 65-0863-14, Thermo Fisher Scientific) to exclude dead cells for 30 min at 4 °C. After washing, the fluorescence intensity of HER3 staining was analyzed using FACSLyric (BD Biosciences, CA, USA) and processed using FlowJo software (FlowJo LLC, Ashland, OR, USA). The relative fluorescence intensity (RFI) was calculated by dividing the median fluorescence intensity of HER3 by that of the isotype control.

### In vitro flow cytometric analysis of HER3 binding specificity

A total of 2 × 10^5^ MDA-MB-468 Luc or MCF-7 cells were harvested and divided into 3 groups: (1) unstained, (2) incubated with 1 µg of HER3-IR700, and (3) pre-blocked with 10-fold molar excess of unconjugated anti-HER3 mAb 30 min before incubation with HER3-IR700. After washing, fluorescence intensity was analyzed using FACSLyric, and the data were processed with FlowJo software.

### In vitro live-cell imaging to monitor NIR-PIT-induced cell death

MDA-MB-468 Luc or MCF-7 cells were seeded at 1 × 10^4^ cells per 35-mm dish and incubated for 24 h. The cells were then incubated with 500 µL of medium containing 2 µg of HER3-IR700 for 1 h at 37 °C. After staining with 8 µg/mL propidium iodide (PI, Cat# P4864, MilliporeSigma, Burlington, MA, USA), the cells were observed using a Leica DM IL LED inverted microscope and a Flexcam C3 (Leica Biosystems, Wetzlar, Germany). Transmitted light differential interference contrast (DIC) and PI fluorescence images were acquired before NIR light irradiation. The cells were subsequently irradiated with NIR light (690 nm, 150 mW/cm^2^, 50 J/cm^2^) using a BWF5 laser system (B&W Tec, Inc., Plainsboro, NJ, USA), and DIC and PI fluorescence images were captured 30 min after laser exposure again.

### In vitro NIR-PIT

MDA-MB-468 Luc or MCF-7 cells were seeded at 1 × 10^5^ cells per well in a 24-well plate in quintuplicate and incubated for 24 h. The cells were then incubated with 500 µL of medium containing 2 µg of HER3-IR700 for 1 h at 37 °C. After changing the medium to a phenol-red-free medium (Cat# 11835030, Thermo Fisher Scientific), the cells were exposed to NIR light (690 nm, 150 mW/cm^2^, 50 J/cm^2^) using an ML7710 laser system (Modulight, Tampere, Finland). The cells wereharvested 1 h later and stained with 1 µg/mL PI to assess the proportion of dead cells using FACSLyric and FlowJo software.

### Animal models

Six- to eight-week-old female athymic nude mice were purchased from Charles River Laboratories (Wilmington, MA, USA). The sample size was determined based on power considerations to ensure adequate statistical validity. Each experimental group included 10 mice, resulting in a total of 20 or 30 mice. With 2 or 3 groups, the resulting *E* value (*E* = total number of animals – number of groups = 20–2 = 18, 30–3 = 27) exceeds the recommended minimum of 10, as suggested by NC3Rs and ARRIVE guidelines [37]. MDA-MB-468 Luc (2 × 10^6^ cells in 100 µL PBS) or MCF-7 (5 × 10^6^ cells in 50 µL Matrigel [Cat# 47743-715, Corning, Corning, NY, USA] + 50 µL PBS) cells were subcutaneously injected into the right dorsum. For MCF-7 models, estradiol valerate (20 µg in 50 µL oil, Cat# 70700-275-22, Hikma Pharmaceuticals, London, UK) was injected intramuscularly into the left thigh once weekly, starting 1 day before inoculation [38]. When the tumor volume reached 50–200 mm^3^, mice were randomized using Tumor Manager (Biopticon, Princeton, NJ, USA) into separate treatment groups. Tumor volumes were determined by the following equation: volume (mm^3^) = length × width^2^ × 0.5 and were measured 1 to 3 times per week by caliper. The mice were euthanized by carbon dioxide inhalation when any of the following endpoints were reached: in the MDA-MB-468 Luc tumor model, when the tumor volume reached 500 mm³, or severe ulceration occurred; and in the MCF-7 tumor model, when the tumor volume reached 2,000 mm³. Mice that survived 74 days after NIR-PIT and had no tumor were defined as a complete response (CR) for this study.

### In vivo NIR-PIT

During the procedure, the mice were anesthetized with inhaled isoflurane (2–3%) and intraperitoneal injection of ketamine (90 mg/kg) and xylazine (10 mg/kg). Tumor-bearing mice were assigned to different experimental groups. In the MDA-MB-468 Luc tumor model, mice were divided into 3 groups: (i) no treatment (Control group); (ii) HER3-IR700-injected (APC group); (iii) NIR-PIT (NIR-PIT group). In the MCF-7 tumor model, mice were divided into 2 groups: (i) no treatment (Control group); (ii) NIR-PIT (NIR-PIT group). Each group consisted of 10 mice. In both the APC and NIR-PIT group, 100 µg of HER3-IR700 was injected intravenously 1 day before NIR-PIT (day − 1), and the tumors in the NIR-PIT group were irradiated with NIR light (690 nm, 150 mW/cm^2^, 50 J/cm^2^) twice, on the following day (day 0) and the day after (day 1). When NIR light was applied, the mice were mostly shielded from light with aluminum foil, except for a small opening to expose the target tumor on the right dorsum. To verify the APC accumulation and photoconversion, IR700 fluorescence images were acquired before and after NIR-PIT using a Pearl Imager. For quantitative analysis, regions of interest were manually drawn to encompass the entire tumor area. For bioluminescence imaging, D-luciferin (15 mg/mL, 200 µL, Cat# LUCK-1G, Gold Biotechnology, St. Louis, MO, USA) was injected intraperitoneally, and the mice were analyzed on a PRISM in vivo imaging system (MediLumine, Montreal, Canada) and Image J (NIH, Bethesda, MD, USA). Regions of Interest were set to include the entire tumor. The signal intensity was shown as photons/cm^2^/sr.

### Flow cytometric analysis of HER3 expression in tumor single-cell suspensions

Tumors that had reached approximately 100 mm³ in volume were excised and enzymatically dissociated using collagenase type IV (1 mg/mL, Cat# LS004188, Worthington Biochemical, Lakewood, NJ, USA) and DNase I (20 µg/mL, Cat# 10104159001, Millipore Sigma). The digested tissues were filtered through a 70-µm cell strainer (Cat# 229484, CELLTREAT Scientific Products, Ayer, MA, USA). The single cells were then stained with the antibodies and dye listed below: anti-CD31 (clone 390, RRID: AB_830757), anti-Podoplanin (clone 8.1.1, RRID: AB_2629802), anti-human HER3, and the corresponding PE-conjugated isotype control (obtained from BioLegend); anti-CD45 (clone 48-0451-82, RRID: AB_1518806), and Fixable Viability Dye (Cat# 65-0866-14, Thermo Fisher Scientific) (obtained from Thermo Fisher Scientific). The stained cells were analyzed using FACSLyric and the data were analyzed with FlowJo software. Dead cells (viability dye positive) were removed from analysis and CD31^−^CD45^−^ Podoplanin^−^ population were defined as tumor cells.

### Histological and immunohistochemical analysis

Tumors were excised from mice in the Control and NIR-PIT groups 1 day after NIR-PIT, and processed into formalin-fixed, paraffin-embedded (FFPE) sections. FFPE sections were stained with hematoxylin and eosin (H&E) for histological evaluation. Immunohistochemistry for HER3 and pan-cytokeratin (pan-CK) was also performed as previously reported, utilizing Opal 6-Plex Detection Kit (RRID: AB_3674065, Akoya Biosciences, Marlborough, MA, USA) and Bond RXm autostainer (Leica Biosystems, Wetzlar, Germany) [39]. Tissue sections were stained with 4’,6-diamidino-2-phenylindole (DAPI) and the antibodies as follows: anti-human HER3 (rabbit poly, RRID: AB_2099571, 1:1000 dilution, Thermo Fisher Scientific), anti-pan-CK (rabbit poly, RRID: AB_10855057, 1:250 dilution; Bioss Antibodies, Woburn, Massachusetts). The stained slides were mounted with ProLong Diamond Antifade Mountant (Cat# P36970, Thermo Fisher Scientific) and analyzed using Mantra Quantitative Pathology Workstation and inForm Tissue Finder software (Akoya Biosciences).

### Statistical analysis

Data are presented as mean ± SEM. Statistical analysis was performed using GraphPad Prism 10.4.1 (GraphPad Software, La Jolla, CA, USA) as described in the figure legends. Statistical significance was set at *p* < 0.05. The adequacy of the sample size was confirmed by post-hoc power analysis using G*Power software 3.1.

## Results

### Verification of IR700 conjugation to anti-HER3 antibody

**Fig. 1:**
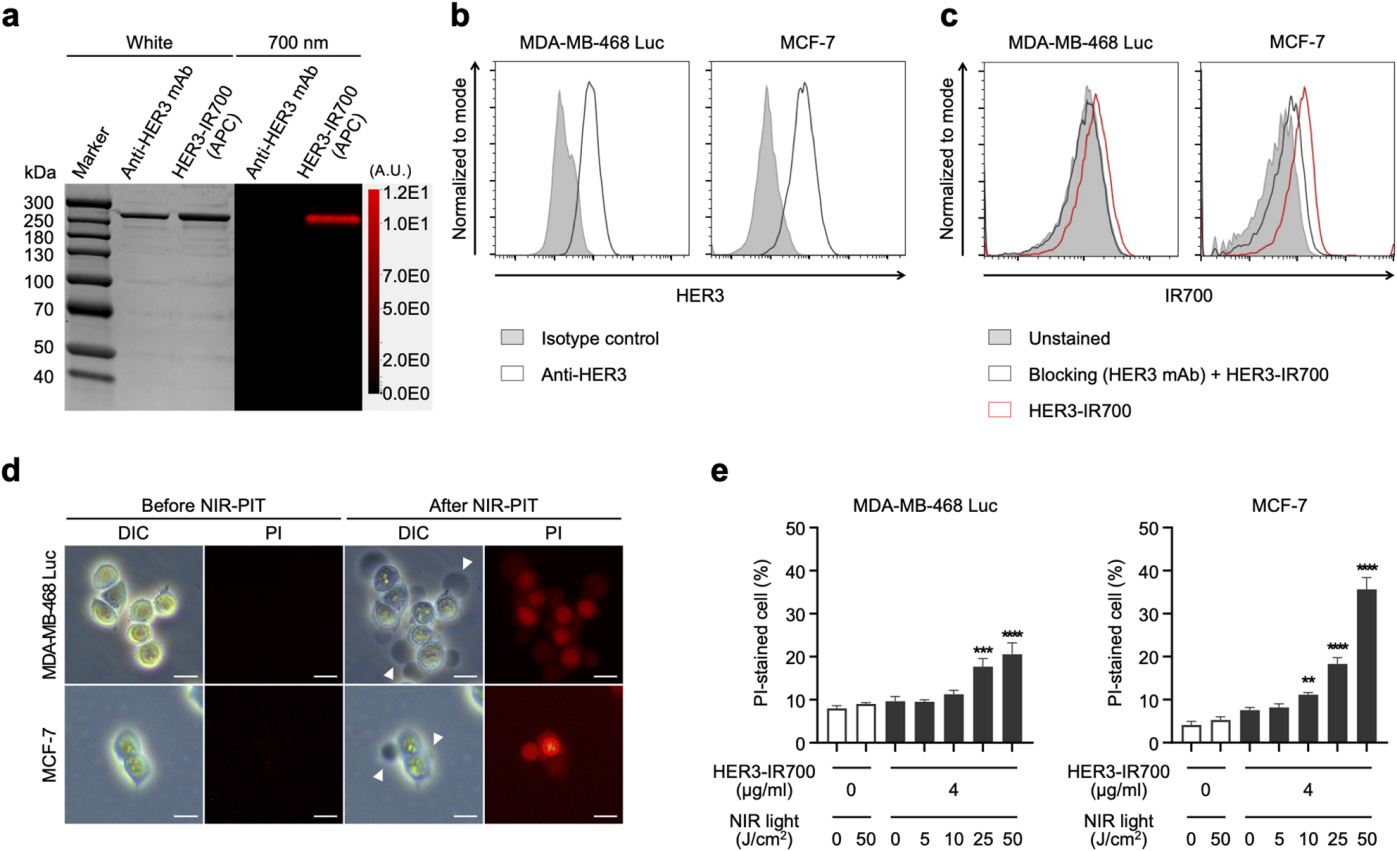
In vitro HER3-targeted NIR-PIT in MDA-MB-468 Luc and MCF-7 cell lines. **a**, SDS-PAGE (left white image: colloidal blue staining. Right red image: 700 nm fluorescence images). The gel was cropped for clarity. Full-length uncropped image is presented in the additional file. **b**, Flow cytometric analysis of HER3 expression on MDA-MB-468 Luc and MCF-7 cell lines. Histograms show fluorescence intensity of HER3 staining (x-axis) and normalized cell counts (y-axis, normalized to mode). Gray-filled histograms represent isotype controls, and white-filled histograms represent HER3 expression on the indicated cell lines. Representative data from three independent experiments are shown. **c**, Flow cytometric analysis of HER3-IR700 binding to MDA-MB-468 Luc cell line. Fluorescence intensity on the x-axis was measured using the APC-Cy7 channel as a surrogate for IR700 signal, and the y-axis represents normalized cell counts (normalized to mode). Gray-filled histograms indicate unstained cells, white-filled histograms indicate blocking with unconjugated anti-HER3 antibody, and a red line represents binding of HER3-IR700. Representative histograms from three independent experiments are shown. **d**, Representative images of cells before and after NIR-PIT on MDA-MB-468 Luc and MCF-7 cell lines, imaged by differential interference contrast (DIC) and propidium iodide (PI) fluorescence microscopy. PI fluorescence is shown in red (Images, ×200; Scale bar, 20 μm). White arrowheads indicate membrane swelling. **e**, Quantification of cell death in MDA-MB-468 Luc and MCF-7 cell lines induced by HER3-targeted NIR-PIT, as assessed by PI staining. The percentage of PI-positive cells (y-axis) represents dead cells. White bars indicate cells without HER3-IR700 treatment, and black bars indicate cells treated with HER3-IR700. Bars are arranged in order of increasing light dose (*n* = 5, mean ± SEM, one-way ANOVA followed by Tukey’s test). **, *p* < 0.01; ***, *p* < 0.001; ****, *p* < 0.0001 vs. untreated control. APC; antibody-photoabsorber conjugate, A.U.; arbitrary units, DIC; transmitted light differential interference contrast, HER3; human epidermal growth factor receptor 3, mAb; monoclonal antibody, NIR: near-infrared, PIT; photoimmunotherapy, PI; propidium iodide

SDS–PAGE analysis was performed to confirm the successful conjugation of HER3-IR700. In the white light image, both the unconjugated anti-HER3 mAb and HER3-IR700 showed bands at similar molecular weights, indicating no apparent change in protein size after conjugation. In contrast, 700 nm fluorescence imaging revealed a clear 700 nm fluorescent signal only in the HER3-IR700 sample, confirming successful labeling of the antibody with IR700 (Fig. 1a).

### In vitro HER3 expression in MDA-MB-468 Luc and MCF-7 cells

Flow cytometry was performed to assess the surface expression of HER3 in MDA-MB-468 Luc and MCF-7 cells. The relative fluorescence intensity (RFI) of HER3 was approximately 4.7 in MDA-MB-468 Luc cells and 8.7 in MCF-7 cells, indicating a higher expression level of HER3 in MCF-7 cells compared with MDA-MB-468 Luc cells (Fig. 1b).

### Specific binding of HER3-IR700 to MDA-MB-468 Luc and MCF-7 cells

To confirm the specific binding of HER3-IR700, MDA-MB-468 Luc and MCF-7 cells were incubated with HER3-IR700 with or without unconjugated anti-HER3 mAb. The fluorescence was observed in the cells with HER3-IR700 alone, whereas these fluorescent signals were blocked by adding an excess of unconjugated anti-HER3 mAb, indicating that HER3-IR700 specifically binds to HER3 on the MDA-MB-468 Luc and MCF-7 cells.

### In vitro therapeutic efficacy of HER3-targeted NIR-PIT

To evaluate the therapeutic effect of HER3-targeted NIR-PIT, MDA-MB-468 Luc and MCF-7 cells were observed using DIC and fluorescence microscopy before and after NIR-PIT. In the DIC images, treated cells showed marked membrane swelling. In the PI fluorescence images, no signal was observed before NIR-PIT, while red fluorescence was detected in the nucleus after NIR-PIT. These findings indicated that HER3-targeted NIR-PIT compromised cell membrane integrity and induced cell death (Fig. 1d). Quantitative analysis using PI staining showed that the percentage of PI-positive cells increased after NIR-PIT in a light dose–dependent manner. Consistent with the expression levels of HER3, MCF-7 cells, which exhibited a higher RFI of HER3 than MDA-MB-468 Luc cells, showed greater sensitivity to the treatment, resulting in a higher proportion of dead cells. In contrast, HER3-IR700 alone and NIR-light irradiation alone did not show a statistically significant increase in PI-positive cells compared with the untreated cells (Fig. 1e).

### HER3 expression in MDA-MB-468 Luc and MCF-7 cells tumor models

**Fig. 2:**
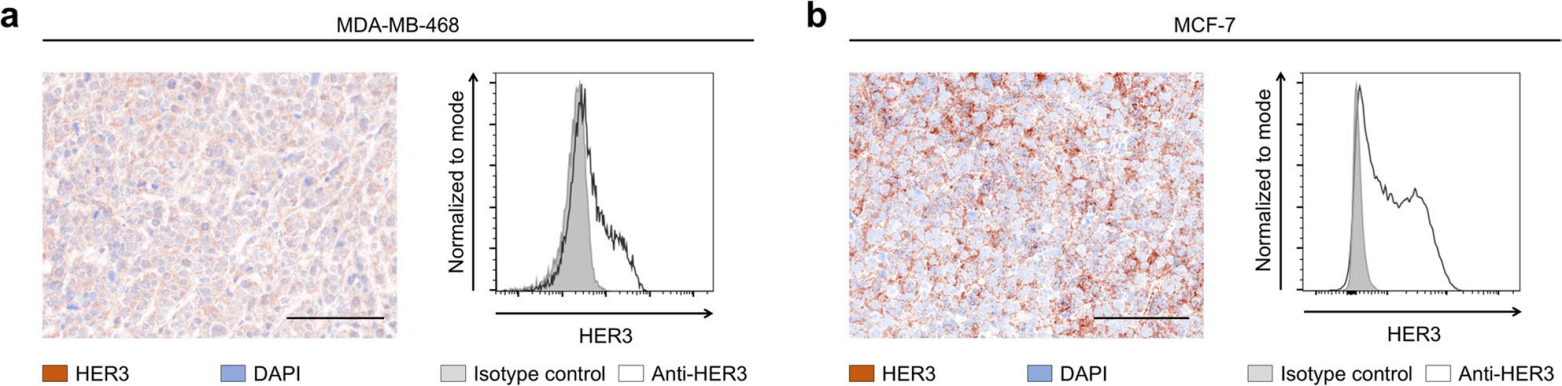
HER3 expression in MDA-MB-468 Luc and MCF-7 tumor mouse models. **a**, **b** Left: Representative immunohistochemistry images are shown (images, ×200; scale bar, 100 μm) in (**a**) MDA-MB-468 Luc and (**b**) MCF-7 tumors. HER3 staining is shown in brown, with nuclei counterstained with DAPI (blue). Right: Flow cytometric analysis of HER3 expression in dissociated tumor cells from (**a**) MDA-MB-468 Luc and (**b**) MCF-7 tumors. Histograms show fluorescence intensity of HER3 staining (x-axis) and normalized mode (y-axis). Gray-filled histograms represent isotype controls, and white-filled histograms represent HER3 expression. Representative data from three independent experiments are shown. DAPI; 4’,6-diamidino-2-phenylindole, HER3; human epidermal growth factor receptor 3

To evaluate the expression of HER3 in MDA-MB-468 Luc and MCF-7 tumors implanted in nude mice, both immunohistochemistry (IHC) and flow cytometric analysis were performed. Immunohistochemical staining of tumor tissues revealed strong HER3 expression in MDA-MB-468 Luc and MCF-7 tumor cells (Fig. 2a and b, left). Consistently, flow cytometry analysis of single-cell suspensions from the tumors confirmed the presence of HER3 on the surface of both tumor cells (Fig. 2a and b, right).

### Therapeutic efficacy of the HER3-targeted NIR-PIT in the MDA-MB-468 Luc tumor model

**Fig. 3:**
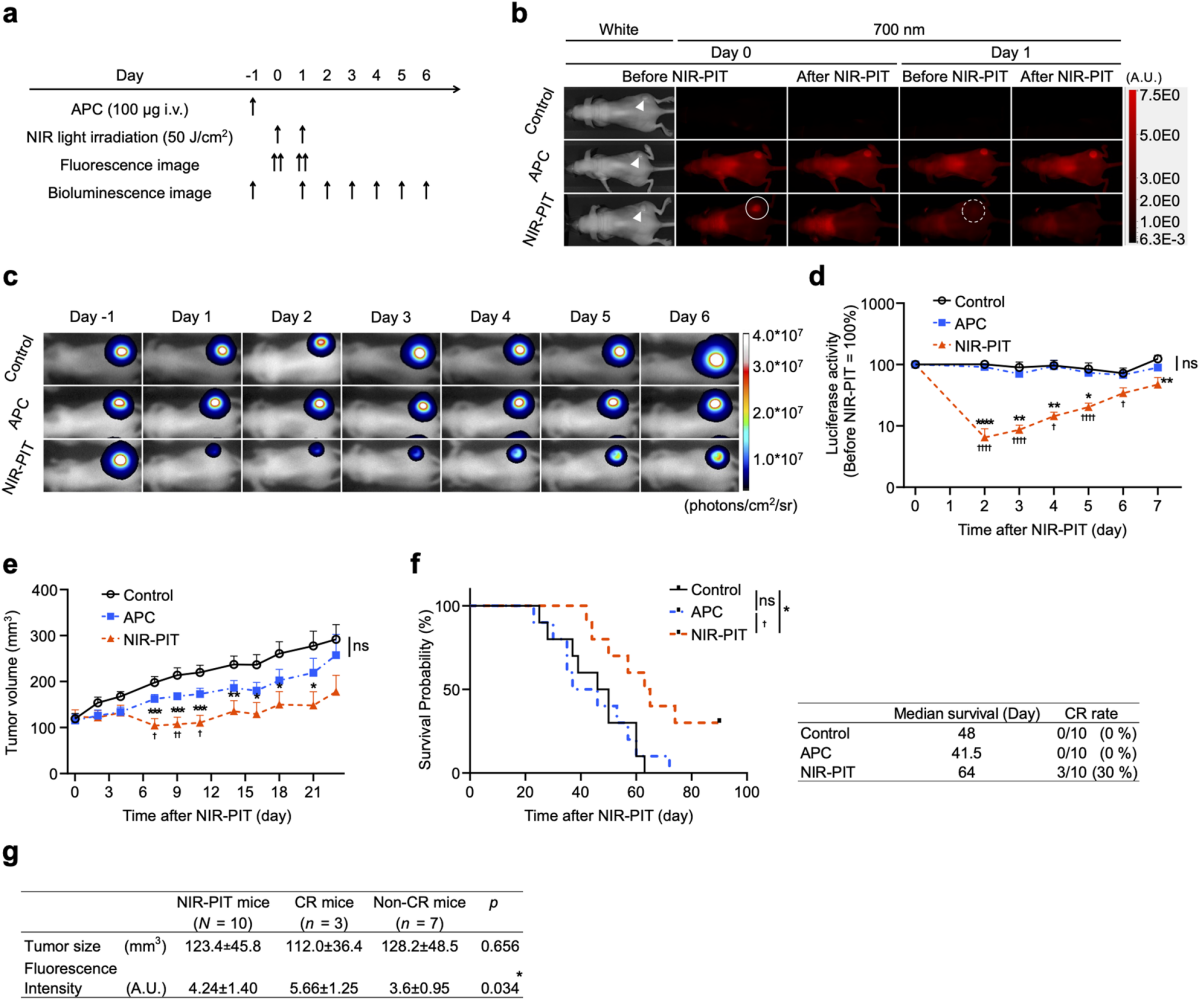
In vivo therapeutic effect of HER3-targeted NIR-PIT for MDA-MB-468 Luc tumor mouse model. Data was obtained from a single experiment. **a**, Treatment schedule. **b**, Representative fluorescent imaging at 700 nm before and after NIR-PIT. Left white images: Bright-field images. Right four red images: 700 nm fluorescence images. White arrowheads indicate the tumors, and white solid and dashed circles denote the accumulation of HER3-IR700 at the tumor sites. **c**, Representative bioluminescence imaging before and after NIR-PIT. **d**, Assessment of Luciferase activity measured using bioluminescence imaging before and after NIR-PIT. Bioluminescence intensity (Y-axis) is shown as a percentage of the Day 0 value (100%) on a logarithmic scale. The X-axis indicates days after treatment (n = 10; mean ± SEM; repeated measures two-way ANOVA followed by Tukey’s test); multiple comparisons. **e**, Tumor growth curves after NIR-PIT. Tumor volume (Y-axis) was measured over time (X-axis, days after treatment); (n = 10; mean ± SEM; repeated measures two-way ANOVA followed by Tukey’s test); multiple comparisons. **f**, Survival curves (n = 10, log-rank test with Bonferroni correction); multiple comparisons, and summary table of survival results. In all graphs, solid line with open circles, dotted line with filled squares, and dashed line with filled triangles represent the control, APC, and NIR-PIT groups, respectively. **g**, Comparison of tumor volumes (mm^3^) and IR700-fluorescence intensities (A.U.) between CR (n = 3) and Non-CR (n = 7) mice groups (Total NIR-PIT mice, N = 10; mean ± SD; unpaired Student’s t-test). All p values in the graphs are expressed as follows: Control vs. NIR-PIT, asterisks (*); APC vs. NIR-PIT, daggers (†); *, †, p < 0.05; **, ††, p < 0.01; ***, p < 0.001; ****, ††††, p < 0.0001; ns, not significant. APC; antibody-photoabsorber conjugate, A.U.; arbitrary units, CR; complete response, HER3; human epidermal growth factor receptor 3, NIR: near-infrared, PIT; photoimmunotherapy, sr; steradian

In the in vivo MDA-MB-468 Luc tumor model, which represents TNBC, the therapeutic efficacy of the HER3-targeted NIR-PIT was evaluated according to the treatment schedule shown in Fig. 3a. Fluorescence imaging confirmed that 700-nm signals accumulated at tumor sites after APC administration. This signal disappeared after NIR light irradiation, confirming IR700 photoactivation. On the following day, faint fluorescence reappeared at the tumor site, suggesting re-accumulation of HER3-IR700. This signal disappeared again after the second NIR light irradiation (Fig. 3b). Bioluminescence imaging was used to assess the cellular activity of cancer cells in the early stage of treatment. Luciferase activity in the NIR-PIT group significantly decreased after NIR-PIT and gradually recovered over time; however, it remained significantly lower than that in the Control and APC injection only (APC) groups (Fig. 3c and d). Tumor volume analysis further showed that only the NIR-PIT group significantly suppressed tumor growth compared with both the Control and APC groups (Fig. 3e). Consistently, survival was also the longest in the NIR-PIT group compared with the other groups, with 30% of the mice achieving CR (Fig. 3f). To investigate the factors contributing to these outcomes, we compared the mice reached CR (CR, *n* = 3) and mice whose tumor size reached endpoint (Non-CR, *n* = 7) subgroups within the NIR-PIT group. While there was no significant difference in baseline tumor volume between the two subgroups, the IR700-fluorescence intensity was significantly higher in the CR mice than in the Non-CR mice (Fig. 3g). These results suggest that HER3-targeted NIR-PIT’s potent antitumor effect against MDA-MB-468 Luc tumors, particularly in mice with higher HER3-IR700 accumulation, which may serve as a predictor of therapeutic success.

### Therapeutic efficacy of the HER3-targeted NIR-PIT in the MCF-7 tumor model

**Fig. 4:**
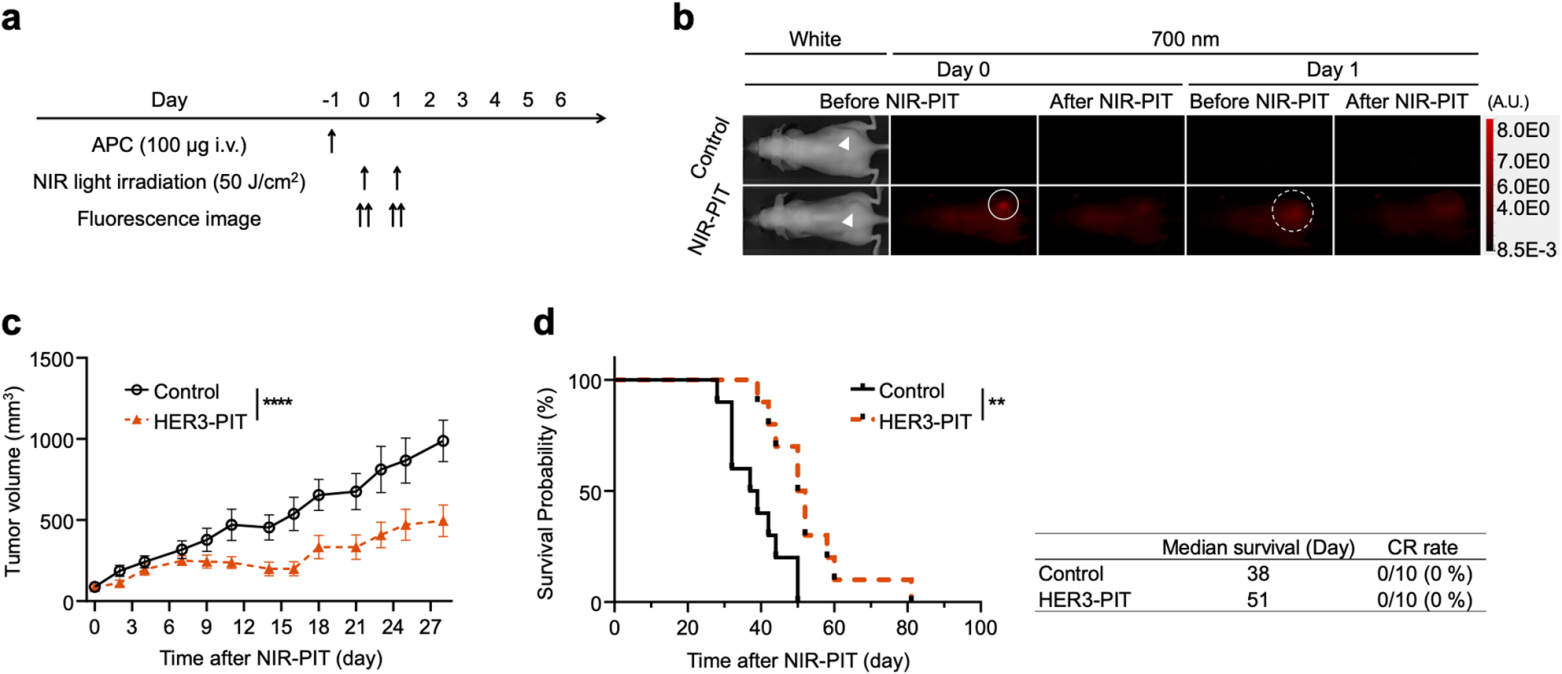
In vivo therapeutic effect of HER3-targeted NIR-PIT for MCF-7 tumor mouse model. Data was obtained from a single experiment. **a**, Treatment schedule. **b**, Representative fluorescent imaging at 700 nm before and after NIR-PIT. Left white images: Bright-field images. Right four red images: 700 nm fluorescence images. White arrowheads indicate the tumors, and white solid and dashed circles denote the accumulation of HER3-IR700 at the tumor sites. **c**, Tumor growth curves after NIR-PIT. Tumor volume (Y-axis) was measured over time (X-axis, days after treatment); (n = 10; mean ± SEM; repeated measures two-way ANOVA focusing on the group × time interaction); ****, p < 0.0001. **d**, Comment Survival curves (n = 10, log-rank test); **, p < 0.01, and summary table of survival results. In all graphs, solid line with open circles and dashed line with filled triangles represent the control and NIR-PIT groups, respectively. APC; antibody-photo absorber conjugate, A.U.; arbitrary units, CR; complete response, HER3; human epidermal growth factor receptor 3, NIR: near-infrared, PIT; photoimmunotherapy

In the in vivo MCF-7 tumor model, which represents luminal breast cancer, the therapeutic efficacy of the HER3-targeted NIR-PIT was evaluated according to the treatment schedule shown in Fig. 4a. Fluorescence imaging demonstrated the accumulation of 700-nm fluorescent signals at tumor sites following APCs administration. This signal disappeared after NIR light irradiation, confirming IR700 photoactivation. On the following day, weak fluorescence reappeared at the tumor site, suggesting re-accumulation of HER3-IR700. This signal disappeared again after the second NIR light irradiation (Fig. 4b). Tumor volume measurements revealed that the NIR-PIT group significantly suppressed tumor growth compared with the Control group (Fig. 4c). Consistently, survival was prolonged in the NIR-PIT group relative to the Control group (Fig. 4d). These results indicate that the HER3-targeted NIR-PIT exhibits antitumor activity against MCF-7 tumors.

### Histopathological changes after HER3-targeted NIR-PIT

**Fig. 5:**
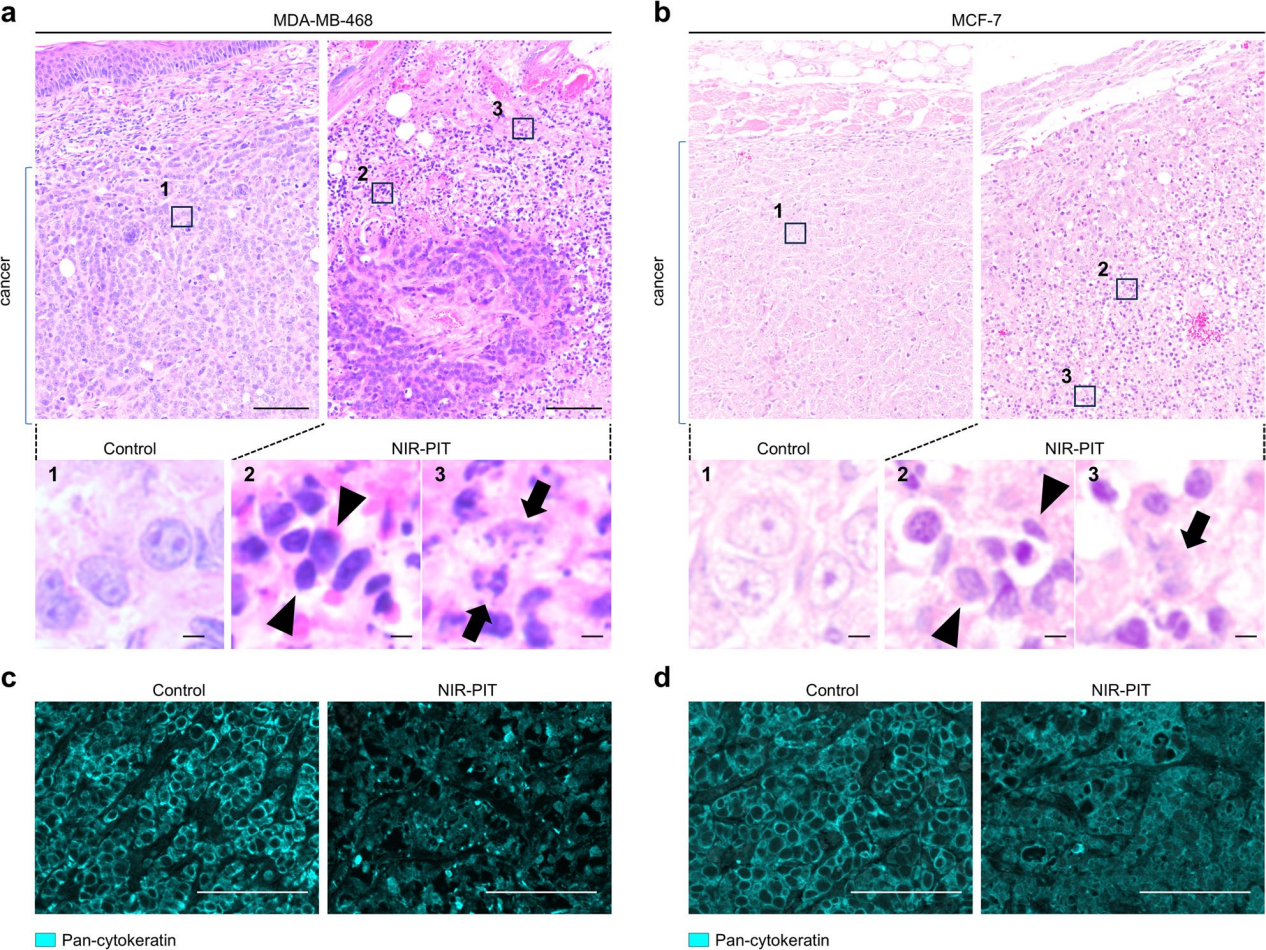
Histological analysis in MDA-MB-468 Luc and MCF-7 tumor mouse models. **a**, **b**, H&E staining of (**a**) MDA-MB-468 Luc and (**b**) MCF-7 tumors 24 h after treatment. Representative images of the control and NIR-PIT groups are shown (images, ×200; scale bar, 100 μm). Enlarged views of the boxed areas are displayed below (scale bar, 10 μm). Arrowheads indicate pyknosis, and arrows indicate karyorrhexis. **c**, **d**, Immunohistochemical staining for pan-cytokeratin in (**c**) MDA-MB-468 Luc and (**d**) MCF-7 tumors 24 h after treatment. Representative images of the control and NIR-PIT groups are shown (images, ×200; scale bar, 100 μm). NIR: near-infrared, PIT; photoimmunotherapy

To evaluate morphological changes, H&E and pan-CK staining were performed. H&E staining of NIR-PIT-treated tumors exhibited cell dissociation and vacuolation, pyknosis, and karyorrhexis (Fig. 5a and b). In the Control, pan-CK was uniformly cytoplasmic, whereas the NIR-PIT group showed blurred and aggregated staining, indicating cytoskeletal disruption (Fig. 5c and d). The observed loss of pan-CK integrity, alongside vacuolation, is suggestive of mechanical cell rupture. These morphological changes are consistent with the unique process induced by NIR-PIT-mediated membrane disruption.

## Discussion

Our findings show that HER3-targeted NIR-PIT has significant antitumor effects against human breast cancer cells expressing HER3, regardless of subtype, both in vitro and in vivo*.* Notably, in vivo HER3-targeted NIR-PIT was effective in TNBC, which has limited treatment options (Figs. 1, 3 and 4).

The remarkable anti-tumor efficacy of HER3-targeted NIR-PIT demonstrated in this study represents a potential breakthrough in overcoming the limitations of conventional signal inhibition therapies. As noted in the Introduction, the clinical efficacy of HER3-targeted monoclonal antibodies has been hindered by the activation of compensatory signaling pathways. However, our in vitro and in vivo analyses confirm that NIR-PIT induces cell death through physical membrane disruption (Figs. 1d and 5). This mechanism suggests that NIR-PIT can reliably eliminate HER3-expressing tumor cells, regardless of any compensatory signal amplification the tumor cells may employ. While HER3 expression in some normal tissues raises concerns about off-tumor toxicity, as seen with HER3-ADC therapies [29, 30], a major advantage of HER3-targeted NIR-PIT is its ability to confine cytotoxicity strictly to the irradiated area [40]. Additionally, since breast cancer typically arises near the body surface, precise light irradiation will further reduce adverse effects. Therefore, NIR-PIT represents a highly promising strategy for targeting HER3, particularly in the context of breast cancer treatment.

Based on our findings, we propose several clinical implications for HER3-targeted NIR-PIT. Among the members of the HER family, therapeutic development has primarily focused on EGFR and HER2. However, their clinical utility in breast cancer is often restricted by subtype-specific expression [35, 41–43]. In this study, we employed two models with contrasting HER-family profiles: MDA-MB-468 Luc (EGFR-positive/HER2-negative) and MCF-7 (EGFR-negative/HER2-positive) [34, 44]. The fact that HER3-targeted NIR-PIT showed significant anti-tumor efficacy in both models suggests that its effectiveness is independent of the status of specific partner receptors, such as EGFR or HER2. This indicates that HER3-targeted NIR-PIT can effectively target breast cancer cells as long as HER3 is expressed. Consequently, such potent efficacy, combined with the frequent expression of HER3 shown in Table 1, adds HER as a target repertoire, making NIR-PIT approaches targeting HER family members more widely applicable. Furthermore, given the correlation between high IR700 fluorescence intensity and superior therapeutic outcomes (Fig. 3g), our data suggest that intratumoral APC accumulation levels—which reflect the interplay between HER3 expression levels and the efficiency of drug delivery to the tumor tissue—could serve as a potential predictive factor for treatment efficacy. These findings suggest the importance of assessing both HER3 expression and APC delivery prior to treatment to maximize the therapeutic benefit. Moreover, HER3 has also been implicated in the development of drug resistance. When EGFR or HER2 signaling is inhibited by targeted therapies, compensatory upregulation of HER3 occurs. This maintains downstream signaling pathways through the heterodimers between activated HER3 and other receptors [45, 46]. Therefore, HER3-targeted NIR-PIT could provide an effective strategy to target tumors that have acquired resistance to EGFR or HER2-targeted therapies. Finally, since HER3 transduces signals through heterodimerization with other HER family members, dual targeting strategies, which utilize either bispecific antibodies or the simultaneous administration of two distinct antibodies targeting HER3 and other HER family members, have been proposed to achieve more effective inhibition of signaling and to overcome drug resistance [3, 5, 47]. On the other hand, NIR-PIT can enhance cytotoxic effects by combining multiple antibody conjugates each targeting a distinct target [48, 49]. Therefore, combining HER3-targeted NIR-PIT with EGFR- or HER2-targeted NIR-PIT may offer an effective strategy to expand therapeutic efficacy against a broader range of breast cancer subtypes.

We acknowledge there are several limitations in this study. First, to evaluate the standalone efficacy of HER3-targeted NIR-PIT, we specifically focused on only two models with contrasting HER-family profiles (EGFR-positive and HER2-positive). While we expect similar effectiveness in HER2-overexpressing models as long as HER3 is expressed, further validation using a wider variety of breast cancer models will be essential to fully confirm the clinical versatility of this approach across the entire breast cancer spectrum. Second, because our in vivo experiments were conducted using immunodeficient mice, the immune-activating effects of HER3-targeted NIR-PIT could not be evaluated. Previous studies have demonstrated that NIR-PIT targeting other antigens can induce a robust anti-tumor immune response [44, 50], thus, similar effects are expected with HER3-targeted NIR-PIT. Third, we could not assess potential side effects. Although anti-HER3 mAbs have already passed phase I/II clinical trials and their safety profiles have been established [3, 51], evaluating the off-tumor effects of NIR-PIT is important. However, testing off-tumor effect in our xenograft models is technically difficult because the human anti-HER3 mAb used in this study does not cross-react with mouse HER3. The cytotoxic activity of NIR-PIT is strictly limited to the area exposed to NIR light [40]; thus, by carefully restricting the irradiation field, off-tumor effects are not expected in clinical settings. Nevertheless, further validation in syngeneic and orthotopic models remains necessary to experimentally confirm the safety of this approach in the presence of surrounding normal mammary tissues. Furthermore, although HER3 is known to be upregulated in tumors resistant to EGFR or HER2 inhibitors, and systematic validation using these resistant models will be beneficial to establish the clinical utility of HER3-targeted NIR-PIT and to translate these findings into effective therapeutic strategies for patients with refractory breast cancer, such studies had to wait until a suitable model becomes available. Finally, antibody selection was not comprehensively explored. Antibodies differ in their epitope specificity to the extracellular domain, ligand-blocking capacity, and internalization efficiency. In this study, we used patritumab, one of the most clinically tested anti-HER3 mAbs. However, patritumab is engineered to enhance internalization [52], which is not optimal for NIR-PIT, which relies on antibody retention on the cell membrane. Even so, our binding assays confirmed sufficient surface binding (Fig. 1c), and we observed clear antitumor efficacy in vivo (Figs. 3 and 4). Therefore, patritumab remains a reasonable candidate for HER3-targeted NIR-PIT, although alternative clones may further improve therapeutic efficacy.

## Conclusions

In conclusion, HER3-targeted NIR-PIT represents an effective therapeutic strategy for HER3-positive breast cancer, providing a widely applicable approach that effective across various subtypes with HER3 expression.

## Supplementary Information

Additional file 1. Original uncropped gel images.

## Data Availability

The data supporting the findings of this study are available from the corresponding author upon reasonable request. A part of the dataset was generated and analyzed using tumor-bearing mouse models. Due to the nature of animal experiments and institutional ethical regulations, raw data are not publicly deposited but can be shared for academic and non-commercial purposes upon request.

## **Abbreviations**

ADCs: Antibody-drug conjugates
APC: Antibody-photoabsorber conjugate
A.U.: Arbitrary units
CR: Complete response
DAPI: 4’,6-diamidino-2-phenylindole
DIC: Transmitted light differential interference contrast
EGFR: Epidermal growth factor receptor
FFPE: Formalin-fixed, paraffin-embedded
H&E: Hematoxylin and eosin
HER: Human epidermal growth factor receptor
IHC: Immunohistochemistry
mAb: Monoclonal antibody
NIR: Near-infrared
Pan-CK: Pan-cytokeratin
PBS: Phosphate-buffered saline
PIT: Photoimmunotherapy
PI: Propidium iodide
RFI: Relative fluorescence intensity
SDS-PAGE: Sodium dodecyl sulfate-polyacrylamide gel electrophoresis
TNBC: Triple-negative breast cancers

## References

[1] Kraus MH, Issing W, Miki T, Popescu NC, Aaronson SA. Isolation and characterization of ERBB3, a third member of the ERBB/epidermal growth factor receptor family: evidence for overexpression in a subset of human mammary tumors. Proc Natl Acad Sci U S A. 1989;86(23):9193–7. 10.1073/pnas.86.23.9193. 2687875 10.1073/pnas.86.23.9193 PMC298460

[2] Plowman GD, Whitney GS, Neubauer MG, Green JM, McDonald VL, Todaro GJ, Shoyab M. Molecular cloning and expression of an additional epidermal growth factor receptor-related gene. Proc Natl Acad Sci U S A. 1990;87(13):4905–9. 10.1073/pnas.87.13.4905. 2164210 10.1073/pnas.87.13.4905 PMC54229

[3] Gandullo-Sánchez L, Ocaña A, Pandiella A HER3 in cancer: from the bench to the bedside. J Exp Clin Cancer Res. 2022;41(1):310. 10.1186/s13046-022-02515-x. 36271429 10.1186/s13046-022-02515-x PMC9585794

[4] Guy PM, Platko JV, Cantley LC, Cerione RA, Carraway KL 3rd. Insect cell-expressed p180erbB3 possesses an impaired tyrosine kinase activity. Proc Natl Acad Sci U S A. 1994;91(17):8132–6. 10.1073/pnas.91.17.8132. 8058768 10.1073/pnas.91.17.8132 PMC44559

[5] Haikala HM, Jänne PA. Thirty Years of HER3: From Basic Biology to Therapeutic Interventions. Clin Cancer Res. 2021;27(13):3528–910. .1158/1078 – 0432.Ccr-20-4465. 33608318 10.1158/1078-0432.CCR-20-4465 PMC8254743

[6] Witton CJ, Reeves JR, Going JJ, Cooke TG, Bartlett JM Expression of the HER1-4 family of receptor tyrosine kinases in breast cancer. J Pathol. 2003;200(3):290. 10.1002/path.1370. 12845624 10.1002/path.1370

[7] Abd El-Rehim DM, Pinder SE, Paish CE, Bell JA, Rampaul RS, Blamey RW, et al Expression and co-expression of the members of the epidermal growth factor receptor (EGFR) family in invasive breast carcinoma. Br J Cancer. 2004;91(8):1532–42. 10.1038/sj.bjc.6602184. 15480434 10.1038/sj.bjc.6602184 PMC2410019

[8] Bae SY, La Choi Y, Kim S, Kim M, Kim J, Jung SP, et al HER3 status by immunohistochemistry is correlated with poor prognosis in hormone receptor-negative breast cancer patients. Breast Cancer Res Treat. 2013;139(3):741–50. 10.1007/s10549-013-2570-6. 23722313 10.1007/s10549-013-2570-6

[9] Berghoff AS, Bartsch R, Preusser M, Ricken G, Steger GG, Bago-Horvath Z, et al. Co-overexpression of HER2/HER3 is a predictor of impaired survival in breast cancer patients. Breast. 2014;23(5):637–43. 10.1016/j.breast.2014.06.011. 25017122 10.1016/j.breast.2014.06.011

[10] Luhtala S, Staff S, Kallioniemi A, Tanner M, Isola J. Clinicopathological and prognostic correlations of HER3 expression and its degradation regulators, NEDD4-1 and NRDP1, in primary breast cancer. BMC Cancer. 2018;18(1):1045. 10.1186/s12885-018-4917-1. 30367623 10.1186/s12885-018-4917-1 PMC6204010

[11] Hassanzadeh Makoui M, Mobini M, Fekri S, Geranpayeh L, Moradi Tabriz H, Madjd Z, et al. Clinico-Pathological and Prognostic Significance of a Combination of Tumor Biomarkers in Iranian Patients With Breast Cancer. Clin Breast Cancer. 2024;24(1):e9. 10.1016/j.clbc.2023.09.013. 37863762 10.1016/j.clbc.2023.09.013

[12] Lee DW, Ryu HS, Nikas IP, Koh J, Kim TY, Kim HK, et al Immune marker expression and prognosis of early breast cancer expressing HER3. Eur J Cancer. 2024;213:115081. 10.1016/j.ejca.2024.115081. 39447449 10.1016/j.ejca.2024.115081

[13] Sinevici N, Edmonds CE, Dontchos BN, Wang G, Lehman CD, Isakoff S, Mahmood U A prospective study of HER3 expression pre and post neoadjuvant therapy of different breast cancer subtypes: implications for HER3 imaging therapy guidance. Breast Cancer Res. 2024;26(1):107. 10.1186/s13058-024-01859-w. 38951909 10.1186/s13058-024-01859-w PMC11218108

[14] Czopek J, Pawlęga J, Fijorek K, Püsküllüoğlu M, Różanowski P, Okoń K HER-3 expression in HER-2-amplified breast carcinoma. Contemp Oncol (Pozn). 2013;17(5):446–9. 10.5114/wo.2013.38564. 24596534 10.5114/wo.2013.38564 PMC3934027

[15] Dębska-Szmich S, Kusińska R, Czernek U, Szydłowska-Pazera K, Habib-Lisik M, Piekarski JH, et al Prognostic value of HER3, PTEN and p-HER2 expression in patients with HER2positive breast cancer. Postepy Hig Med Dosw (Online). 2015;69:586–97. 10.5604/17322693.1151339. 25983297 10.5604/17322693.1151339

[16] Ho-Pun-Cheung A, Bazin H, Boissière-Michot F, Mollevi C, Simony-Lafontaine J, Landas E, et al Quantification of HER1, HER2 and HER3 by time-resolved Förster resonance energy transfer in FFPE triple-negative breast cancer samples. Br J Cancer. 2020;122(3):397–404. 10.1038/s41416-019-0670-8. 31792349 10.1038/s41416-019-0670-8 PMC7000684

[17] Lyu H, Shen F, Ruan S, Tan C, Zhou J, Thor AD, Liu B HER3 functions as an effective therapeutic target in triple negative breast cancer to potentiate the antitumor activity of gefitinib and paclitaxel. Cancer Cell Int. 2023;23(1):204. 10.1186/s12935-023-03055-w. 37716943 10.1186/s12935-023-03055-w PMC10504712

[18] Shi F, Telesco SE, Liu Y, Radhakrishnan R, Lemmon MA ErbB3/HER3 intracellular domain is competent to bind ATP and catalyze autophosphorylation. Proc Natl Acad Sci U S A. 2010;107(17):7692–7. 10.1073/pnas.1002753107. 20351256 10.1073/pnas.1002753107 PMC2867849

[19] Majumder A. HER3: Toward the Prognostic Significance, Therapeutic Potential, Current Challenges, and Future Therapeutics in Different Types of Cancer. Cells. 2023;12(21):103390cells12212517. 10.3390/cells12212517 PMC10648638 37947595

[20] Mirschberger C, Schiller CB, Schraml M, Dimoudis N, Friess T, Gerdes CA, et al. RG7116, a therapeutic antibody that binds the inactive HER3 receptor and is optimized for immune effector activation. Cancer Res. 2013;73:5183–94. 10.1158/0008-5472.CAN-12-2868. 23780344 10.1158/0008-5472.CAN-13-0099

[21] Hong M, Yoo Y, Kim M, Kim JY, Cha JS, Choi MK, et al. A Novel Therapeutic Anti-ErbB3, ISU104 Exhibits Potent Antitumorigenic Activity by Inhibiting Ligand Binding and ErbB3 Heterodimerization. Mol Cancer Ther. 2021;20(6):1142–52. 10.1158/1535-7163.MCT-20-0907. 33782100 10.1158/1535-7163.MCT-20-0907

[22] Turowec JP, Lau EWT, Wang X, Brown KR, Fellouse FA, Jawanda KK, et al. Functional genomic characterization of a synthetic anti-HER3 antibody reveals a role for ubiquitination by RNF41 in the anti-proliferative response. J Biol Chem. 2019;294:1396–409. 10.1074/jbc.RA118.004420. 30523157 10.1074/jbc.RA118.004420 PMC6349115

[23] D’Souza JW, Reddy S, Goldsmith LE, Shchaveleva I, Marks JD, Litwin S, et al. Combining anti-ERBB3 antibodies specific for domain I and domain III enhances the anti-tumor activity over the individual monoclonal antibodies. PLoS ONE. 2014;9(11):e112376. 10.1371/journal.pone.0112376. 25386657 10.1371/journal.pone.0112376 PMC4227695

[24] Jacobsen HJ, Poulsen TT, Dahlman A, Kjaer I, Koefoed K, Sen JW, et al. Pan-HER, an Antibody Mixture Simultaneously Targeting EGFR, HER2, and HER3, Effectively Overcomes Tumor Heterogeneity and Plasticity. Clin Cancer Res. 2015;21(18):4110–22. 10.1158/1078-0432.CCR-14-3312. 25908781 10.1158/1078-0432.CCR-14-3312

[25] Geuijen CAW, De Nardis C, Maussang D, Rovers E, Gallenne T, Hendriks LJA, et al. Unbiased Combinatorial Screening Identifies a Bispecific IgG1 that Potently Inhibits HER3 Signaling via HER2-Guided Ligand Blockade. Cancer Cell. 2018;33(5):922–e3610. 10.1016/j.ccell.2018.04.003. 29763625 10.1016/j.ccell.2018.04.003

[26] Schaefer G, Haber L, Crocker LM, Shia S, Shao L, Dowbenko D, et al. A two-in-one antibody against HER3 and EGFR has superior inhibitory activity compared with monospecific antibodies. Cancer Cell. 2011;20(4):472–86. 10.1016/j.ccell.2011.09.003. 22014573 10.1016/j.ccr.2011.09.003

[27] Campbell MR, Ruiz-Saenz A, Zhang Y, Peterson E, Steri V, Oeffinger J, et al Extensive conformational and physical plasticity protects HER2-HER3 tumorigenic signaling. Cell Rep. 2022;38(5):110285. 10.1016/j.celrep.2021.110285. 35108526 10.1016/j.celrep.2021.110285 PMC8865943

[28] Hashimoto Y, Koyama K, Kamai Y, Hirotani K, Ogitani Y, Zembutsu A, et al. A Novel HER3-Targeting Antibody-Drug Conjugate, U3-1402, Exhibits Potent Therapeutic Efficacy through the Delivery of Cytotoxic Payload by Efficient Internalization. Clin Cancer Res. 2019;25(23):7151–61 10.1158/1078-0432.Ccr-19-1745. 31471314 10.1158/1078-0432.CCR-19-1745

[29] Jänne PA, Baik C, Su WC, Johnson ML, Hayashi H, Nishio M et al. Efficacy and Safety of Patritumab Deruxtecan (HER3-DXd) in EGFR Inhibitor-Resistant, EGFR-Mutated Non-Small Cell Lung Cancer. Cancer Discov. 2022;12(1):74-89 10.1158/2159-8290.Cd-21-0715. 10.1158/2159-8290.CD-21-0715 PMC9401524 34548309

[30] Krop IE, Masuda N, Mukohara T, Takahashi S, Nakayama T, Inoue K, et al Patritumab Deruxtecan (HER3-DXd), a Human Epidermal Growth Factor Receptor 3-Directed Antibody-Drug Conjugate, in Patients With Previously Treated Human Epidermal Growth Factor Receptor 3-Expressing Metastatic Breast Cancer: A Multicenter, Phase I/II Trial. J Clin Oncol. 2023;41(36):5550–60. 10.1200/jco.23.00882. 37801674 10.1200/JCO.23.00882 PMC10730028

[31] Kobayashi H, Choyke PL Near-Infrared Photoimmunotherapy of Cancer. Acc Chem Res. 2019;52(8):2332–9. 10.1021/acs.accounts.9b00273. 31335117 10.1021/acs.accounts.9b00273 PMC6704485

[32] Mitsunaga M, Ogawa M, Kosaka N, Rosenblum LT, Choyke PL, Kobayashi H Cancer cell-selective in vivo near infrared photoimmunotherapy targeting specific membrane molecules. Nat Med. 2011;17(12):1685–91. 10.1038/nm.2554. 22057348 10.1038/nm.2554 PMC3233641

[33] Furusawa A, Choyke PL, Kobayashi H NIR-PIT: Will it become a standard cancer treatment? Front Oncol. 2022;12:1008162. 10.3389/fonc.2022.1008162. 36185287 10.3389/fonc.2022.1008162 PMC9523356

[34] Nagaya T, Sato K, Harada T, Nakamura Y, Choyke PL, Kobayashi H Near Infrared Photoimmunotherapy Targeting EGFR Positive Triple Negative Breast Cancer: Optimizing the Conjugate-Light Regimen. PLoS ONE. 2015;10(8):e0136829. 10.1371/journal.pone.0136829. 26313651 10.1371/journal.pone.0136829 PMC4552472

[35] Yamashita S, Kojima M, Onda N, Yoshida T, Shibutani M Trastuzumab-based near-infrared photoimmunotherapy in xenograft mouse of breast cancer. Cancer Med. 2023;12(4):4579–89. 10.1002/cam4.5302. 36259134 10.1002/cam4.5302 PMC9972010

[36] Okada R, Maruoka Y, Furusawa A, Inagaki F, Nagaya T, Fujimura D, et al The Effect of Antibody Fragments on CD25 Targeted Regulatory T Cell Near-Infrared Photoimmunotherapy. Bioconjug Chem. 2019;30(10):2624–33. 10.1021/acs.bioconjchem.9b00547. 31498995 10.1021/acs.bioconjchem.9b00547 PMC7413076

[37] Charan J, Kantharia ND How to calculate sample size in animal studies? J Pharmacol Pharmacother. 2013;4(4):303–6. 10.4103/0976-500x.119726. 24250214 10.4103/0976-500X.119726 PMC3826013

[38] Behzadi R, Fattahi S, Momtaz MR, Kavoosian S, Asouri M, Akhavan-Niaki H. Injectable Estradiol Valerate, as a Substitute for Estradiol Pellets in Breast Cancer Animal Model. Int Biol Biomedical J. 2015;1(1):35–8.

[39] Kato T, Okada R, Furusawa A, Inagaki F, Wakiyama H, Furumoto H et al. Simultaneously Combined Cancer Cell- and CTLA4-Targeted NIR-PIT Causes a Synergistic Treatment Effect in Syngeneic Mouse Models. Mol Cancer Ther. 2021;20(11):2262-73 10.1158/1535-7163.Mct-21-0470. 10.1158/1535-7163.MCT-21-0470 PMC10214494 34518299

[40] Mitsunaga M, Nakajima T, Sano K, Kramer-Marek G, Choyke PL, Kobayashi H Immediate in vivo target-specific cancer cell death after near infrared photoimmunotherapy. BMC Cancer. 2012;12:345. 10.1186/1471-2407-12-345. 22873679 10.1186/1471-2407-12-345 PMC3502522

[41] Miyazaki NL, Furusawa A, Choyke PL, Kobayashi H. Review of RM-1929 Near-Infrared Photoimmunotherapy Clinical Efficacy for Unresectable and/or Recurrent Head and Neck Squamous Cell Carcinoma. Cancers (Basel). 2023;15(21):103390cancers15215117. 10.3390/cancers15215117 PMC10650558 37958293

[42] Li X, Zhao L, Chen C, Nie J, Jiao B Can EGFR be a therapeutic target in breast cancer? Biochim Biophys Acta Rev Cancer. 2022;1877(5):188789. 10.1016/j.bbcan.2022.188789. 36064121 10.1016/j.bbcan.2022.188789

[43] Morrison MM, Hutchinson K, Williams MM, Stanford JC, Balko JM, Young C, et al ErbB3 downregulation enhances luminal breast tumor response to antiestrogens. J Clin Invest. 2013;123(10):4329–43. 10.1172/jci66764. 23999432 10.1172/JCI66764 PMC3784526

[44] Furusawa A, Takao S, Suzuki M, Kano M, Yamamoto H, Kano M, et al. EpCAM-targeted near-infrared photoimmunotherapy (NIR-PIT) for the treatment of breast cancer. Ann Med. 2025;57(1):2540599. 40792441 10.1080/07853890.2025.2540599 PMC12344674

[45] Sergina NV, Rausch M, Wang D, Blair J, Hann B, Shokat KM, Moasser MM Escape from HER-family tyrosine kinase inhibitor therapy by the kinase-inactive HER3. Nature. 2007;445(7126):437–41. 10.1038/nature05474. 17206155 10.1038/nature05474 PMC3025857

[46] Narayan M, Wilken JA, Harris LN, Baron AT, Kimbler KD, Maihle NJ Trastuzumab-induced HER reprogramming in resistant breast carcinoma cells. Cancer Res. 2009;69(6):2191–4. 10.1158/0008-5472.Can-08-1056. 19276389 10.1158/0008-5472.CAN-08-1056

[47] Uberall I, Krízová K, Steigerová J. Cetuximab enhances the anti-proliferative effect of trastuzumab in ERBB2 over-expressing breast cancer cells–preliminary study. Klin Onkol. 2011;24(5):356–60. 22070017

[48] Nakajima T, Sano K, Choyke PL, Kobayashi H Improving the efficacy of Photoimmunotherapy (PIT) using a cocktail of antibody conjugates in a multiple antigen tumor model. Theranostics. 2013;3(6):357–65. 10.7150/thno.5908. 23781283 10.7150/thno.5908 PMC3677407

[49] Siddiqui MR, Railkar R, Sanford T, Crooks DR, Eckhaus MA, Haines D et al Targeting Epidermal Growth Factor Receptor (EGFR) and Human Epidermal Growth Factor Receptor 2 (HER2) Expressing Bladder Cancer Using Combination Photoimmunotherapy (PIT). Sci Rep. 2019;9(1):2084. 10.1038/s41598-019-38575-x. 10.1038/s41598-019-38575-x PMC6375935 30765854

[50] Nagaya T, Friedman J, Maruoka Y, Ogata F, Okuyama S, Clavijo PE, et al. Host immunity following near-infrared photoimmunotherapy is enhanced with PD-1 checkpoint blockade to eradicate established antigenic tumors. Cancer Immunol Res. 2019;7(3):401–13. 10.1158/2326-6066.CIR-18-0546. 30683733 10.1158/2326-6066.CIR-18-0546 PMC8237708

[51] Nishio M, Horiike A, Murakami H, Yamamoto N, Kaneda H, Nakagawa K, et al Phase I study of the HER3-targeted antibody patritumab (U3-1287) combined with erlotinib in Japanese patients with non-small cell lung cancer. Lung Cancer. 2015;88(3):275–81. 10.1016/j.lungcan.2015.03.010. 25891541 10.1016/j.lungcan.2015.03.010

[52] Malm M, Frejd FY, Ståhl S, Löfblom J Targeting HER3 using mono- and bispecific antibodies or alternative scaffolds. MAbs. 2016;8(7):1195–209. 10.1080/19420862.2016.1212147. 27532938 10.1080/19420862.2016.1212147 PMC5058629

