# Supplementary material for: Human epidermal growth factor receptor 3-targeted near-infrared photoimmunotherapy in a xenograft mouse model of breast cancer
Source: BMC Cancer. 2026 May 18;26:832. doi: 10.1186/s12885-026-16170-4 (PMC13349167; doi:10.1186/s12885-026-16170-4)
Supplement: Supplementary file 1 — Additional file 1. Original uncropped gel images. [file 12885_2026_16170_MOESM1_ESM.pdf]

Additional file 1

Full-length original images of SDS-PAGE and IR700 fluorescence imaging.

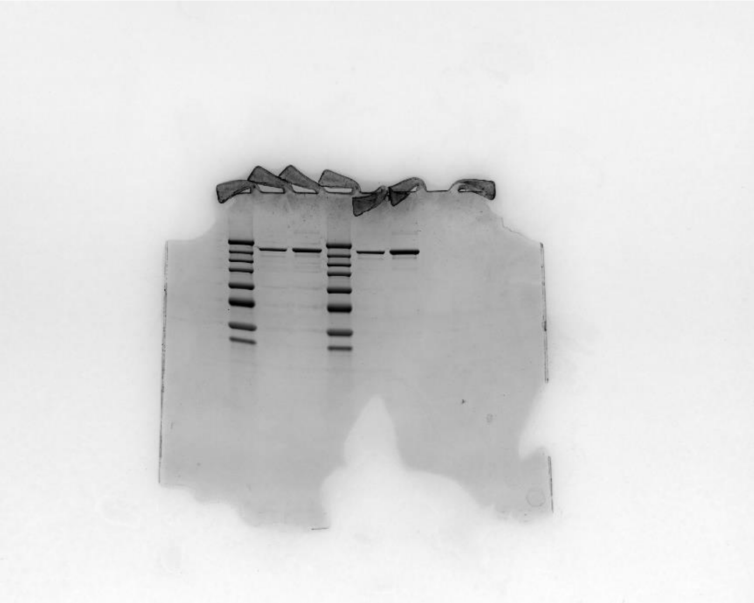

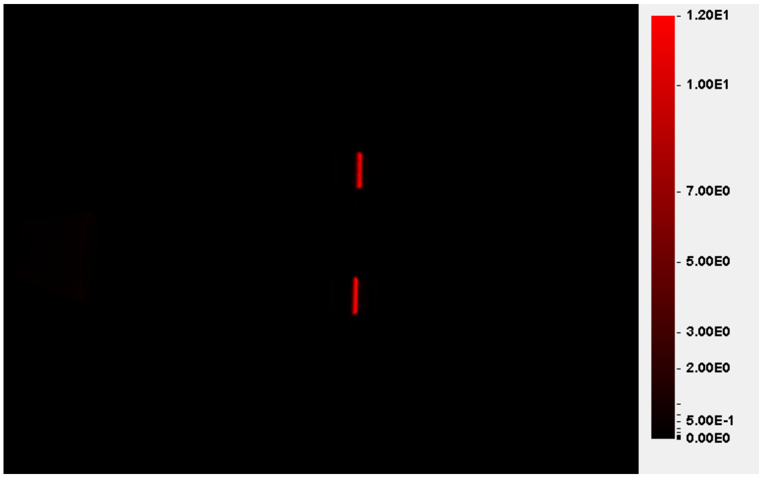

The top panel shows the original SDS-PAGE gel image captured with a Bio-Rad ChemiDoc MP imaging system. The lanes from left to right are: (1) molecular weight marker, (2) unconjugated anti-HER3 monoclonal antibody, (3) HER3-APC, (4) molecular weight marker, (5) unconjugated another monoclonal antibody, and (6) another monoclonal antibody-APC. The bottom panel shows the fluorescence imaging of the same gel captured with a Pearl imager (700 nm channel), rotated 90 degrees clockwise from the top image. Red signals indicate the accumulation of IR700 in the conjugated antibody lanes.
